# A TCF-Based Carbon Monoxide NIR-Probe without the Interference of BSA and Its Application in Living Cells

**DOI:** 10.3390/molecules27134155

**Published:** 2022-06-28

**Authors:** Yingxu Wu, Xiaojing Deng, Lan Ye, Wei Zhang, Hu Xu, Boyu Zhang

**Affiliations:** 1 College of Medical Laboratory, Dalian Medical University, Dalian 116044, China; wuyingux1111@gmail.com (Y.W.); dengxiaojing0728@hotmail.com (X.D.); 2 Advanced Institute for Medical Sciences, Dalian Medical University, Dalian 116044, China; 15606072750@163.com; 3 Department of Spine Surgery, The Second Hospital of Dalian Medical University, Dalian 116023, China; zhangwei1983@dmu.edu.cn

**Keywords:** carbon monoxide, Tsuji-Trost reaction, fluorescent probe, bovine serum albumin (BSA)

## Abstract

As toxic gaseous pollution, carbon monoxide (CO) plays an essential role in many pathological and physiological processes, well-known as the third gasotransmitter. Owning to the reducibility of CO, the Pd^0^-mediated Tsuji-Trost reaction has drawn much attention in CO detection in vitro and in vivo, using allyl ester and allyl ether caged fluorophores as probes and PdCl_2_ as co-probes. Because of its higher decaging reactivity than allyl ether in the Pd^0^-mediated Tsuji-Trost reaction, the allyl ester group is more popular in CO probe design. However, during the application of allyl ester caged probes, it was found that bovine serum albumin (BSA) in the fetal bovine serum (FBS), an irreplaceable nutrient in cell culture media, could hydrolyze the allyl ester bond, and thus give erroneous imaging results. In this work, dicyanomethylenedihydrofuran (TCF) and dicyanoisophorone (DCI) were selected as electron acceptors for constructing near-infrared-emission fluorophores with electron donor phenolic OH. An allyl ester and allyl ether group were installed onto TCF-OH and DCI-OH, constructing four potential CO fluorescent probes, TCF-ester, TCF-ether, DCI-ester, and DCI-ether. Our data revealed that ester bonds of TCF-ester and DCI-ester could completely hydrolyze in 20 min, but ether bonds in TCF-ether and DCI-ether tolerate the hydrolysis of BSA and no released fluorescence was observed even up to 2 h. Moreover, passing through the screen, it was concluded that TCF-ether is superior to DCI-ether due to its higher reactivity in a Pd^0^-mediated Tsuji-Trost reaction. Also, the large stokes shift of TCF-OH, absorption and emission at 408 nm and 618 nm respectively, make TCF-ether desirable for fluorescent imaging because of differentiating signals from the excitation light source. Lastly, TCF-ether has been successfully applied to the detection of CO in H9C2 cells.

## 1. Introduction

Carbon monoxide (CO) is widely known as a “silent killer” and major air pollution. It has a higher ability to bind to the heme iron centers in hemoglobin and cytochrome P450 in mitochondria of up to 500 times that of oxygen. The US Environmental Protection Agency (EPA) has set two national health protection standards for CO with 35 and 9 ppm over 1 and 8 h, respectively. When a preternormal concentration of CO is inhaled by mammals, it can lead to fatal consequences to health, or even to death [1,2,3]. However, recent studies have indicated that this “silent killer” can be produced constantly in the mammalian body during the metabolism of heme and heme oxygenase [4,5,6,7]. As a signaling molecule like NO and H_2_S, CO has been demonstrated to play vital roles in both pathological and physiological processes, including but not limited to anti-inflammatory responses [8,9,10], anti-oxidation, anti-apoptosis [9], vascular smooth muscle [11,12,13], vasorelaxation [14], cellular proliferation and apoptotic [15,16], and in Alzheimer’s disease [17]. Therefore, developing new tools to detect CO in the living system is of great importance.

Some methods have been applied to detect CO including gas chromatography, colorimetric assays [18,19,20], electrochemical assays [21,22], etc. These techniques are unsuitable for real-time tracking of CO in live biological samples in a noninvasive manner, which limits their further development in the study of the biological function of CO. Compared with the above-mentioned methods, fluorescence-based imaging techniques have drawn worldwide attention. Numerous advantages, such as its nondestructive quality, convenience, deep tissue penetration, high sensitivity, and spatiotemporal resolution, render it irreplaceable for detecting intracellular biological molecules in living systems [23,24,25]. A number of fluorescent probes for the specific detection of CO have been proposed [26,27,28,29,30,31]. Chang et al. reported the first organic palladium complexes’ fluorescent probes (COP-1) to detect endogenous CO in 2012 [32]. After this, Tang et al. developed two BODIPY-Pd complex probes ACP-1 and ACP-2 [6]. Wilton-Ely and Martínez-Máñez’s group reported a series of Ru(II), Rh(II), and Os(II) vinyl complexes as probes for detecting CO with high sensitivity [33,34,35]. However, obvious drawbacks persist in these two types of metal complex-based probes such as strenuous synthesis, weak stability, photobleaching, and high background fluorescence.

The Tsuji-Trost reaction was first reported by Tsuji et al. in 1965 and was further developed by Trost et al. in 1973 [36]. In this reaction, the palladium catalyst first coordinates with a substrate that contains a leaving group (fluorophore) in an allylic position, then undergoes oxidative addition, forming the π-allyl complex. This allyl complex can then be attacked by a nucleophile, resulting in the activation of fluorescence [37,38]. Since Siddhartha Pal et al. developed a Pd(0)-mediated intramolecular cyclization-elimination reaction to track CO in biosystems, several fluorescent probes based on this mechanism have been published [39,40,41,42,43,44,45], and most of these reported that the molecular probes of CO used allyl carbonate or allyl carbamate as the reaction site. It is noteworthy that some researchers have shown that the albumin has the esterase-like activities and this character is ingeniously used in the activation of prodrugs [46,47,48]. Fetal bovine serum (FBS) is widely used as a growth supplement for the in vitro cell culture of eukaryotic cells, in which the concentration of albumin (specifically referred to as bovine serum albumin, BSA) is in the range of 20–36 mg/mL. Both facts might point to a likelihood that allyl carbonate-based CO probes can’t be used in the in vitro cell research. Furthermore, human serum albumin (HSA) has identical esterase-like activities which help keep colloidal osmolality in the bloodstream. The tolerability to albumin is a significant parameter in the development of in-vivo or clinical CO probes. Recently, a number of probes have been reported for the sensitive and selective detection of BSA or HSA, among which the ester group is one of the classic alternatives [49,50,51]. Hong et al. developed activity-based fluorescent probes for detecting HSA in blood-contaminated tissue samples on the basis of its pseudo-esterase activity using an acyl moiety as a reaction-site [52]. The 4-biphenylcarboxylic ester group was screened out as to have excellent selectivity and sensitivity towards HSA over a variety of hydrolases as installed on commercially available 1,8-naphthalimide and acridinone derivatives [50,51]. Furthermore, Ahn’s group reported two exceptional works in which they found that ester-group-based probes have a risk of reacting with esterase in vivo in the course of the development of an NAD(P)H quinone oxidoreductase-1 probe and a cysteine probe [53,54]. Recently, in a study on resorufin-based probes responsive to nitroreductase, the probe of p-nitrobenzyl-resorufin linked with carbonate exhibited strongly fluorescent turn-on behavior as incubated with BSA, indicating that esterase-like activity of BSA or HSA is an essential consideration in the probes’ design [55].

**Scheme 1:**
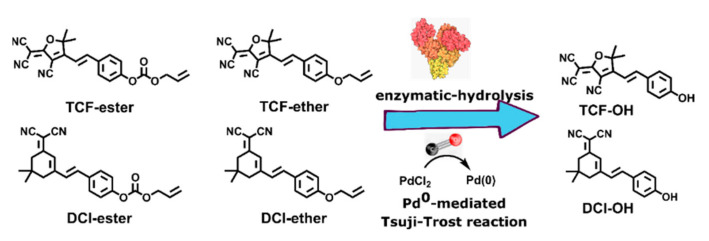
The enzymatic-hydrolysis vs Pd^0^-mediated Tsuji-Trost reaction.

In this contribution, dicyanomethylenedihydrofuran (TCF) and dicyanoisophorone (DCI) were selected as an electron-withdrawing group for constructing near-infrared-emission fluorophores with electron donor phenolic OH. An allyl ester and allyl ether group were installed onto TCF-OH and DCI-OH, constructing four potential CO fluorescent probes, TCF-ester, TCF-ether, DCI-ester, and DCI-ether (Scheme 1). TCF moiety as a strong intramolecular charge transfer (ICT) electron-withdrawing group has been widely used for the preparation of nonlinear optical materials [56,57], single-molecule imaging [58], and activated localization microscopy [59]. DCI moiety as an electron-withdrawing group in ICT probe possesses strong NIR fluorescence with a large Stokes shift over 100 nm and is widely applied in specific light-up bioprobes [60]. In this work, the competitive relationships between the enzymatic-hydrolysis and the Pd^0^ mediated Tsuji-Trost reaction for probes of TCF-ester, TCF-ether, DCI-ester, and DCI-ether were systematically investigated to guide the applications of such probes in the medical research field.

## 2. Results and Discussions

### 2.1. Design and Synthesis of the Fluorescent Probe DCI-ether and TCF-ether

**Scheme 2:**
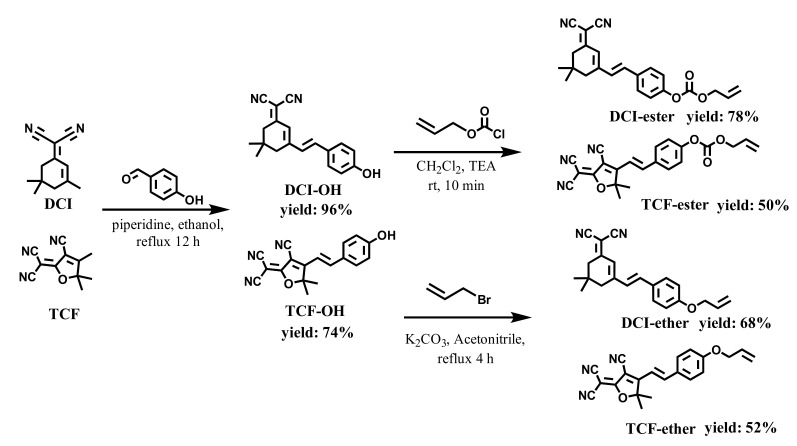
The synthesis routes of DCI-ester, TCF-ester, DCI-ether, and TCF-ether.

Firstly, DCI-ester and TCF-ester were synthesized using the literature procedure by coupling p-Hydroxybenzaldehyde to DCI and TCF, and then the allyl ester group was installed onto the hydroxy group of DCI-OH and TCF-OH, respectively, to obtain DCI-ester and TCF-ester. [30,61,62]. They were tested to verify the hydrolysis of probes in the presence of BSA. Actually, most CO probes based on the Tsuji-Trost reaction contain allyl ester as the reaction site; these ester bonds on probes can be hydrolyzed by BSA to produce fluorescent molecules (TCF-OH and DCI-OH) with ICT effects, causing an inevitable interference in the imaging of CO. To overcome this obstacle, we develop two fluorescent probes, DCI-ether and TCF-ether, in which the ether group was selected as a substitute for the allyl ester group. The synthetic route of probe TCF-ester, TCF-ether, DCI-ester, and DCI-ether is shown in Scheme 2. The synthetic procedure and compound characterization can be found in the Supplementary Materials.

### 2.2. Spectral Properties and the Sensing Mechanism

**Figure 1:**
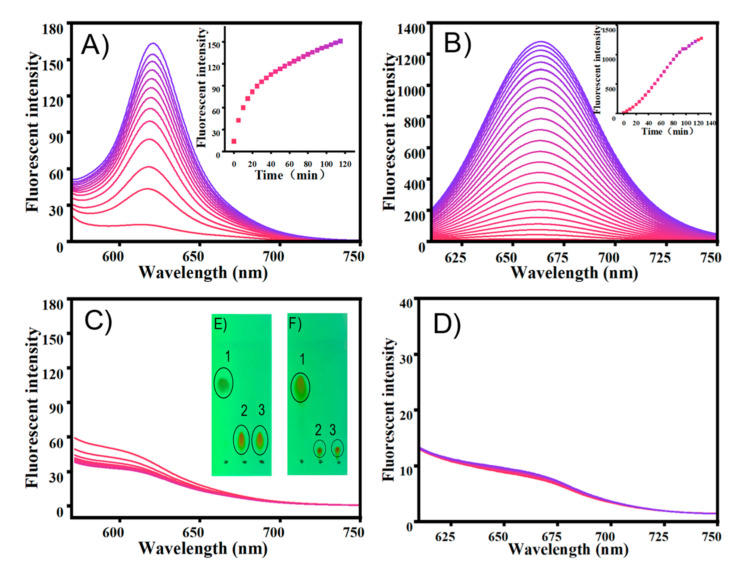
The emission changes of probe (**A**) TCF-ester, λ_ex_ = 580 nm, (**B**) DCI-ester, λ_ex_ = 560 nm, (**C**) TCF-ether and (**D**) DCI-ether with BSA (23 mg/mL) in PBS buffer (10 mM, pH 7.4, containing 30% DMSO, *v/v*) at 37 °C for 2 h. (**E**) Sample spotted in the TLC plates: 1. DCI, 2. DCI + BSA, 3. DCI-OH. (**F**) Sample spotted in the TLC plates: 1. TCF-ester, 2. TCF-ester + BSA, 3. TCF-OH. Color changes (0–2 h), from pink to bule.

**Figure 2:**
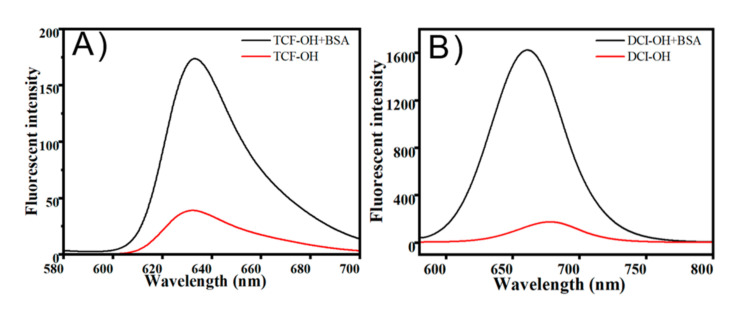
(**A**) Fluorescence spectra of TCF-OH, TCF-OH + BSA in PBS buffer (10 mM, pH 7.4, containing 30% DMSO, *v/v*) at 37 °C. (**B**) Fluorescence spectra of DCI-OH, DCI-OH + BSA in PBS buffer (10 mM, pH 7.4, containing 30% DMSO, *v/v*) at 37 °C.

Firstly, the fluorescent change of TCF-ester and DCI-ester were tested in the presence of BSA. As shown in Figure 1A, TCF-ester (50 μM) was incubated in a PBS buffer solution (10 mM, pH 7.4 containing 30% DMSO, *v/v*) at 37 °C in the presence of BSA (23 mg/mL), and the fluorescence intensity at 618 nm gradually enhanced within 2 h and continued to grow. Akin to TCF-ester, the probe DCI-ester incubated with BSA exhibited a roughly similar increment of fluorescence intensity at 666 nm (Figure 1B). It corroborated BSA to be effective in the hydrolysis of probes (Figure 1E,F). Furthermore, compared to the probe TCF-ester and DCI-ester, the effect of BSA on the fluorescence of the as-synthesized TCF-ether and DCI-ether could be neglected (Figure 1C,D). These results are consistent with the hypothesis that the ester could be hydrolyzed by BSA, which may lead to the misjudgment of imaging, and the hydrolysis might be avoided by using an allyl ether cage for the Tsuji-Trost reaction. Most interestingly, the plateaus of fluorescent intensity of DCI-ester and TCF-ester in BSA are higher than the fluorescent intensity of DCI-OH and TCF-OH in PBS (Figure 2). As shown in Figure 2A, TCF-OH showed a single emission band at 632 nm upon the excitation at 580 nm. After adding 7 × 10^−3^ eqiv. BSA to those solutions and immediately testing its fluorescent spectra, there was five times enhancement in fluorescence intensity for TCF-OH. DCI-OH showed a characteristic emission band at 677 nm (Figure 2B), the same as TCF-OH, and the emission intensity of DCI-OH was substantially enhanced upon adding BSA, but the emission peak was blue shifted from 677 nm to 661 nm.

**Figure 3:**
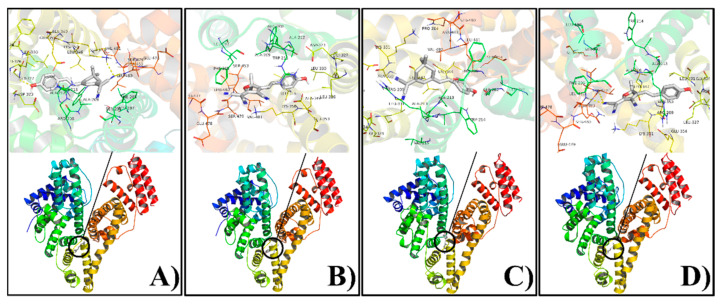
Molecular docking of DCI-OH(**A**,**C**) and TCF-OH(**B**,**D**) with albumins (**A**,**B** for BSA; **C**,**D** for HSA). Upper: amino acid residues involved; Lower: cartoon model representing DCI-OH and TCF-OH as stick while HSA and BSA are represented by ribbon model.

It has been demonstrated that the fluorophore with hydrophobic characteristics tends to selectively bind to site II of BSA, a hydrophobic environment that reduces the occurrence of aggregation-caused quenching (ACQ) and reinforces the ICT effect. To find the cause of the fluorescence enhancement in Figure 2 and verify this intuition, we performed molecular docking studies to confirm the experimental results for the BSA-TCF-OH/DCI-OH interaction. The results showed that TCF-OH and DCI-OH interact with site II on BSA as well as on HSA. The best energy ranked results and the amino acid residues involved in the interaction are shown in Figure 3 and summarized in Table 1. DCI-OH positively fits into the hydrophobic compartment to sub domain IIIA in Sudlow site II of BSA and HSA, with ΔG values of −8.3 kcal·mol^−1^ and −9.3 kcal·mol^−1^ respectively. Meanwhile, TCF-OH docks in the sub domain IIIA of Sudlow site II of BSA and HSA with ΔG values of −6.2 kcal·mol^−1^ and −8.7 kcal·mol^−1^, respectively. DCI-OH interacts hydrophobically with Ser201, Phe205, Arg208, Ala209, Ala212, Trp213, Asp323, Leu326, Phe329, Leu330, Ser343, Leu346, Glu353, and Ser453 residues of BSA near site II, whereas Gly327, Lys350 and Leu480 form hydrogen bonds with DCI-OH. On the contrary, Leu198, Ser202, Phe206, Ala210, Ala213, Trp214, Val216, Asp324, Leu327, Gly328, Leu331, Val343, Leu347, Ala350, Lys351, Glu354, Pro384, Ser454, Ser480, Leu481, Val482 and Asn483 present in the site of HSA interacts with DCI-OH hydrophobically, and Arg209 residues are involved in hydrogen bond formation at site II of HAS. To TCF-OH, the amino acid residues of BSA Leu197, Gly327, Ala 349, Lys350, Thr477, Glu478, Ser479, Leu480, and Val 481 are involved in hydrophobic interaction in comparison to DCI-OH. No hydrogen bond is formed between TCF-OH and BSA. Three amino acid residues of HSA Arg209, Thr478, and Glu479 interact with TCF-OH hydrophobically, and another three amino acid residues Asp324, Leu481, and Val482 interact with TCF-OH through hydrogen bonds in comparison to DCI-OH. The binding free energy of Sudlow site I with DCI-OH and TCF-OH was also calculated, obtaining −5.8 kcal·mol^−1^ and −4.3 kcal·mol^−1^ for BSA and −5.2 kcal·mol^−1^ and −7.0 kcal·mol^−1^ for HSA. Those data showed that both DCI-OH and TCF-OH preferably dock in sub domain IIIA of Sudlow site II of BSA and HSA through hydrophobic interaction. Thus, the dramatic increase in the fluorescence intensities of TCF-OH and DCI-OH in BSA can be attributed to the docking in sub domain IIIA in Sudlow site II of BSA and HSA. Furthermore, from a report on the reaction of human albumin with aspirin in vitro, it can be inferred that DCI-ester and TCF-ester stably acetylate lysines and release DCI-OH and TCF-OH, and then enter the hydrophobic domain of BSA or HSA [63].

**Figure 4:**
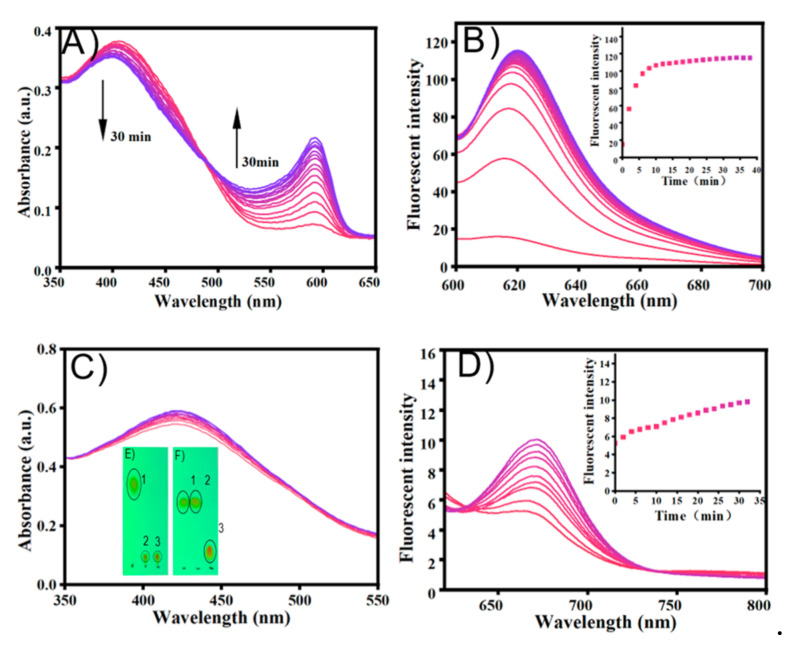
Absorption spectra of probe (**A**) TCF-ether (50 μM) and (**C**) DCI-ether (50 μM) in the presence of PdCl_2_ (50 μM) upon addition of CORM-2 (100 μM) in PBS buffer (10 mM, pH 7.4, with 30% DMSO, *v/v*) at 37 °C for 30 min. Fluorescence spectra changes of probe (**B**) TCF-ether and (**D**) DCI-ether in the presence of PdCl_2_ (50 μM) upon addition of CORM-2 (100 μM). Inset: Time-dependent fluorescence intensity of TCF-ether and DCI-ether. (**E**) Sample spotted in the TLC plates: 1. TCF-ether, 2. TCF-ether + CORM-2, 3. TCF-OH. (**F**) Sample spotted in the TLC plates: 1. DCI-ether, 2. DCI-ether + CORM-2, 3. DCI-OH. TCF-ether: λ_ex_ = 580 nm, DCI-ether: λ_ex_ = 560 nm. Color changes (0–2 h), from pink to bule.

Next, the sensing properties of TCF-ether and DCI-ether in the presence of Pd^2+^ for CO were implemented in a PBS buffer (10 mM, pH 7.4, containing 30% DMSO, *v/v*) at 37 °C. A carbon monoxide releasing molecule, [Ru_2_Cl_4_(CO)_6_] (CORM-2), was used as a source for carbon monoxide. TCF-ether showed strong absorption at 408 nm and almost no fluorescence emissions. After CORM-2 was added, the absorption at 408 nm gradually decreased and a new absorption peak appeared at 592 nm. At the same time, a gradually rising fluorescence emission at 618 nm was detected, reaching a stable state after around 12 min (Figure 4A,B). However, the changes of probe DCI-ether in the spectra of absorption and emission are relatively inconspicuous. As shown in Figure 4C,D, the probe DCI-ether showed an absorption maximum at 410 nm and almost no fluorescence in the NIR region; upon the addition of CORM-2, it showed a slight change in the absorption spectrum and the fluorescence emission at 666 nm also barely increased. To understand the sensing mechanism, we employed TLC to confirm the final products, and as Figure 4E,F showed, the reaction generated fluorescein TCF-OH as the final products and no DCI-OH was produced. The above experiments indicate that the TCF-ether probe could serve as a promising tool to detect CO in further applications with remarkable fluorescence enhancement. Unfortunately, the DCI-ether probe displayed a lower reaction activity compared to TCF-ether, and the change in its spectrum is almost negligible.

### 2.3. TCF-Ether Response to Different Concentrations of CO

**Figure 5:**
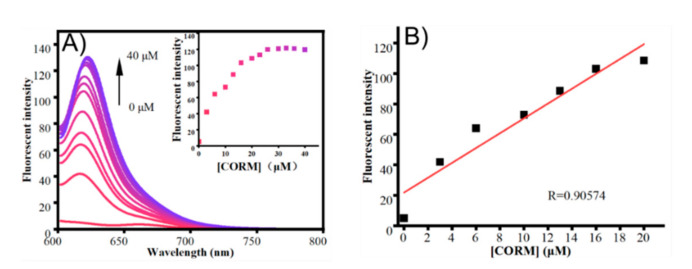
(**A**) Fluorescent spectra changes of probe TCF-ether (50 μM) in the presence of PdCl_2_ (50 μM) upon the addition of different concentrations of CORM-2 in PBS buffer (10 mM, pH 7.4, with 30% DMSO, *v/v*) at 37 °C. Insert: fluorescence intensity changes of the probe system at 618 nm as a function of the concentrations of CORM-2. (**B**) Linear correlation between emission intensity and concentration of CORM-2 (0–20 μM) at 618 nm. Each spectrum was obtained 15 min after mixing. λ_ex_ = 580 nm.

To further investigate the performance of the probe system toward various concentrations of CO, CORM-2 was added to a PBS buffer in the presence of PdCl_2_. With the addition of CORM-2 (0–40 μM), the emission peak at 618 nm gradually increased, and saturation reached about 15 min (Figure 5). Furthermore, the fluorescence intensity at 618 nm exhibited an excellent linear relationship in the range of 0–20 μM (R = 0.90574). These results demonstrate that TCF-ether can rapidly and fluorescently detect CO with significantly enhanced fluorescence intensity.

### 2.4. Selectivity to CO

**Figure 6:**
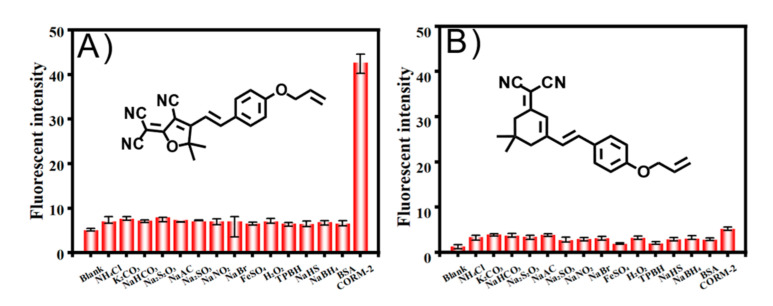
Fluorescent intensity responses of the probe system (**A**) TCF-ether and (**B**) DCI-ether in the presence of PdCl_2_ (50 μM) for various analytes (100 μM, except 20 μM of CORM-2). TCF-ether: λ_ex_ = 580 nm, DCI-ether: λ_ex_ = 560 nm.

The selectivity of these new CO-detection systems (TCF-ether and DCI-ether) was then investigated in a PBS buffer solution. As shown in Figure 6, various competitive analytes such as NH₄Cl, K₂CO₃, NaHCO₃, Na₂S₂O₃, NaAC, Na₂SO₃, NaNO₂, NaBr, FeSO₄, H₂O₂, TPBH, NaHS, NaBH₄, and BSA (23 mg/mL), were added to the probe solution, respectively. Among these analytes, for TCF-ether, only 20 μM CORM-2 induced a significant fluorescence enhancement, while the other analytes showed almost no fluorescence changes. However, there was little fluorescence intensity increase when the DCI-ether was treated with CORM-2 (Figure 6B). These results clearly imply that the TCF-ether probe, compared to DCI-ether, possesses reasonable selectivity to CO.

### 2.5. pH Study of Probes

**Figure 7:**
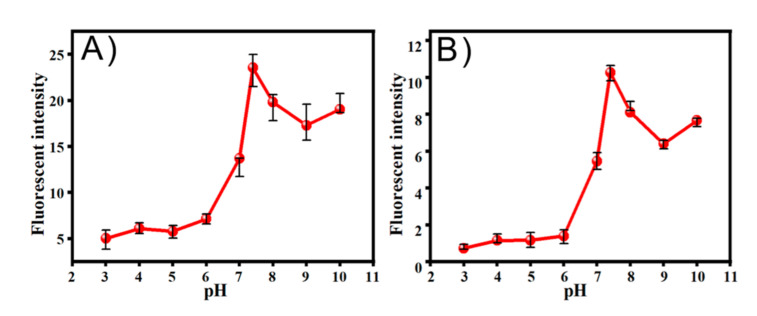
The effects of pH values on the fluorescence intensity of (**A**) TCF-ether and (**B**) DCI-ether.

Next, the performance of TCF-ether and DCI-ether in the presence of CO toward different pH environments was explored. Probe TCF-ether system appeared almost no emission within the pH range from 3 to 6, and a significant fluorescence response can be observed in the range of 7 to 10 (Figure 7A). Meanwhile, only a weak fluorescence was detected at pH 7.4 in the DCI-ether system (Figure 7B). Under acidic conditions, the negative charge of the phenolic hydroxyl group will be protonated, and protonation will affect the process of intramolecular charge transfer, eventually leading to a decrease in fluorescence intensity. These results indicate that TCF-ether is better at detecting CO under physiological conditions.

### 2.6. The Bioimaging Applications

**Figure 8:**
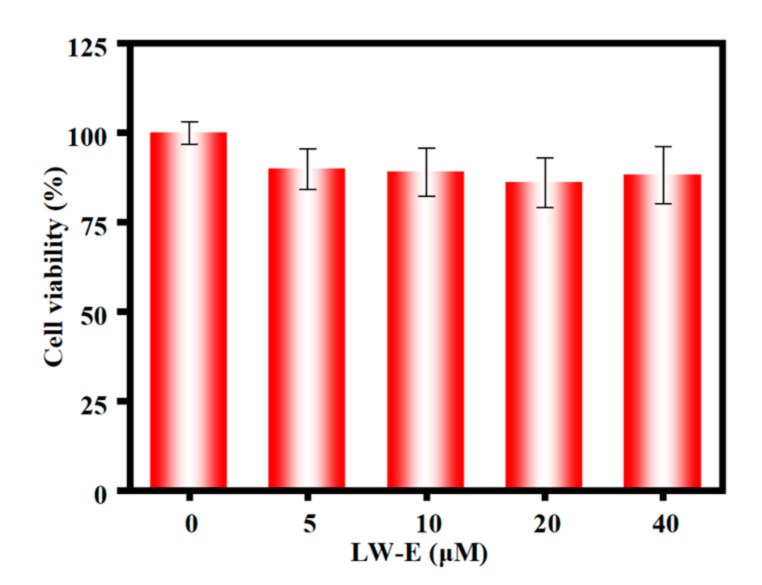
Cytotoxicity assay of H9C2 in the presence of various concentrations of TCF-ether from 0 to 40 µM for 4 h.

On the basis of the inspiring spectral performances shown above, we investigated the potential applications of TCF-ether for imaging CO in living cells. H9C2 cells, a cardiac myoblast, was selected because CO can alleviate ventricular fibrillation (VF) induced by ischemia-reperfusion [64]. Carbon monoxide is becoming significant candidate for the treatment of heart disease. The cytotoxicity of TCF-ether and TCF-ether + PdCl_2_ was first explored by CCK8 assays, and the results indicate that both TCF-ether (up to 40 μM) and the TCF-ether + PdCl_2_ system showed low cytotoxicity to H9C2 cells (Figure 8), thus it can be used for cell imaging observation.

**Figure 9:**
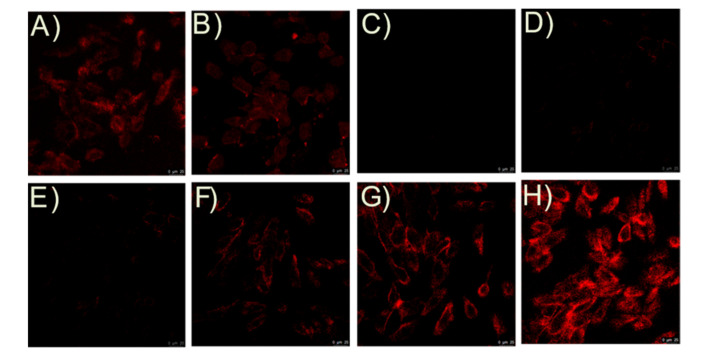
Fluorescence images of H9C2 with TCF-ester and TCF-ether. Cells were incubated with TCF-ester in the (**A**) presence or (**B**) absence of BSA (23 mg/mL). Cells were incubated with TCF-ether in the (**C**) presence or (**D**) absence of BSA (23 mg/mL). Cells were pre-incubated with the probe system for 30 min and then incubated with CORM-2 (**E**) 0 µM, (**F**) 5 µM, (**G**) 20 µM, and (**H**) 40 µM, for another 30 min at 37°C. Scale bars, 25 μM.

Then, the bioimaging applications of probe TCF-ether were performed. We first verified the anti-interference properties toward BSA of the TCF-ether compared with TCF-ester. H9C2 cells were incubated with 10 µM probe (TCF-ether, TCF-ester) in Dulbecco’s Modified Eagle’s Medium (DMEM, Gibco, Grand Island, NY, USA) supplemented with or without BSA (23 mg/mL) at 37 °C in a 5% CO_2_ incubator for 2 h. As shown in Figure 9A, probe TCF-ester exhibits a red fluorescence in the cells that incubated with BSA. Surprisingly, when the cells were incubated in DMEM without any BSA, a red emission can still be observed, presumably due to the proteins in cells that have esterase-like activity (Figure 9B). Nevertheless, probe TCF-ether engenders no fluorescence emission in cells whether incubated in BSA or BSA-free culture medium (Figure 9C,D). These results indicate that TCF-ether exhibits superior performance in anti-interference properties toward BSA compared to TCF-ester.

Next, H9C2 cells were incubated with TCF-ether (10 µM) for 30 min at 37 °C, then treated with PdCl_2_ (10 µM). After 30 min, no fluorescence signals in the red channel were observed, indicating the probe system barely showed background fluorescence. In contrast, when the cells were pre-incubated with probe system for 30 min, then with CORM-2 for another 30 min, distinct enhancement of red fluorescence in H9C2 cells appeared (Figure 9E–H), and the fluorescence was dose-dependent to CORM-2. These results demonstrate that the TCF-ether is applicable to image exogenous CO in living cells.

## 3. Materials and Methods

All reagents and chemicals were purchased from commercial suppliers and used without further purification. Ultrapure water was prepared through the Sartorious Arium 611DI system and used throughout the experiments. Thin-layer chromatography (TLC) analysis was performed on silica gel plates (F254, Merck KGaA (Darmstadt, Germany)). Silica gel (200–300 mesh, Qingdao Haiyang Chemical Co. (Qingdao, China)) was used for column chromatography. All NMR data were taken on a 400 MHz spectrometer (Bruker Co., Lt.d, Germany (Darmstadt, Germany)). All chemical shifts are reported in ppm values using the peak of TMS as an internal reference. Fluorescence emission spectra were obtained on a HITACHI-F4700 spectrophotometer. Absorbance spectra were recorded on a SP-2500 UV−vis spectrophotometer (Shanghai spectrum). Fluorescence images were acquired by a Leica TCS-SP8 SR confocal microscope, and images were generated using ImageJ software.

### 3.1. Synthesis and Characterization

Compound DCI-ester, DCI-OH, TCF-OH and TCF-ester are synthesized according to the published methods [61,65]. The synthetic procedure and compound characterization can be found in the Supplementary Materials.

#### 3.1.1. Synthesis of DCI-ether

DCI-OH (100 mg, 0.34 mmol) was completely dissolved in 5 mL of acetonitrile solution. And then cesium carbonates (337 mg, 1.03 mmol) was added to the solution. The mixture was stirred thoroughly, allyl chloride (125 mg, 1.03 mmol) was added to the solution, and refluxed for 4 h. The reaction mixture was then concentrated under reduced pressure to give a crude solid, which was purified by silica gel column chromatography (petroleum ether: ethyl acetate = 10:1, *v/v*) to afford an orange solid (140 mg, 68%). ^1^H NMR (400 MHz, DMSO-d6) δ 7.76–7.68 (m, 2H), 7.33 (s, 2H), 7.11–7.01 (m, 2H), 6.90 (s, 1H), 6.11 (ddt, J = 17.4, 10.5, 5.2 Hz, 1H), 5.47 (dq, J = 17.2, 1.8 Hz, 1H), 5.34 (dq, J = 10.5, 1.6 Hz, 1H), 4.68 (dt, J = 5.3, 1.7 Hz, 2H), 2.67 (s, 2H), 2.60 (s, 2H), 1.08 (s, 6H). ^13^C NMR (101 MHz, DMSO) δ 170.75, 159.98, 156.85, 138.08, 133.88, 130.06, 129.22, 127.77, 122.31, 118.14, 115.59, 114.49, 113.68, 75.82, 68.76, 42.79, 38.65, 32.13, 27.91. 

#### 3.1.2. Synthesis of TCF-ether

TCF-OH (100 mg, 0.33 mmol) was completely dissolved in 10 mL of acetonitrile solution. And then cesium carbonates (323 mg, 0.99 mmol) was added to the solution. The mixture was stirred thoroughly, allyl chloride (120 mg, 0.99 mmol) was added to the solution, and refluxed for 4 h. The reaction mixture was then concentrated under reduced pressure to give a crude solid, which was purified by silica gel column chromatography (petroleum ether: ethyl acetate = 3:1, *v/v*) to afford an orange solid (60 mg, 52%). ^1^H NMR (400 MHz, DMSO-d6) δ 8.01–7.90 (m, 2H), 7.92–7.76 (m, 1H), 7.18–7.07 (m, 3H), 6.10 (ddt, J = 16.2, 10.5, 5.2 Hz, 1H), 5.46 (dt, J = 17.3, 1.7 Hz, 1H), 5.34 (dt, J = 10.6, 2.0 Hz, 1H), 4.76–4.67 (m, 2H), 1.83 (s, 3H), 1.70 (s, 3H). ^13^C NMR (101 MHz, DMSO) δ 177.87, 177.66, 176.12, 166.88, 162.36, 161.59, 148.08, 145.81, 133.62, 133.51, 132.30, 131.57, 127.69, 127.65, 118.44, 118.34, 116.04, 115.85, 113.63, 113.48, 113.35, 113.30, 112.47, 111.55, 99.64, 98.01, 96.14, 88.11, 69.03, 68.92, 54.15, 25.71, 25.59.

### 3.2. Optical Studies

The stock solutions of probe (5 mM), dichlorotricarbonylruthenium (II) dimer (CORM-2, 1 mM), and PdCl_2_ (5 mM) were prepared in DMSO. CORM-2 has been confirmed as an easy-to-handle CO source, and thus it was chosen to serve as the CO releasing component in this work; it should be used when it is fresh. All spectral tests were carried out in a DMSO-PBS buffer solution (10 mM, pH 7.4 containing 30% DMSO, v/v). The concentration of each analyte was 100 μM, except for BSA (23 mg/mL) and CORM-2 (20 μM). The fluorescence spectra were collected with λ_ex_ = 550 nm and 560 nm, with a slit width *d*_ex_ = *d*_em_ = 10 nm. 

### 3.3. Cell Viability Assays

H9C2 cells were prepared for viability studied in 96-well plates and maintained at 37 °C in a 5% CO_2_ incubator for 24 h. Then the substrate was replaced with Dulbecco’s Modified Eagle’s Medium (DMEM, Invitrogen) basic medium, and different concentrations of probe (10 μM) were added to the 96-well plate. The cells were treated with CCK-8 (cell-counting Kit-8) at 37 °C for 4 h. The absorbance at 450 nm was measured using a microplate reader (TransGen Biotechnology, China). The cellular viability of each group was determined by defining the absorbance of the controls group to be 100% viable.

### 3.4. Cell Culture and Imaging of Exogenous CO

The H9C2 cells were cultured in DMEM, supplemented with 10% FBS (Hyclone) in an atmosphere of 5% CO_2_ at 37 °C. In the control experiment, the cells were incubated with TCF-ether solution (10 μM) and a mixture of TCF-ether (10 μM) and PdCl_2_ (10 μM), respectively, for 30 min at 37 °C. They were then washed with 1 mL of PBS three times, and then the fluorescence images were obtained. 

Two groups of H9C2 cells were carried out by different treatments in order to confirm the anti-interference of probes. The first group was treated with TCF-ester (10 μM) in DMEM, which was added with or without BSA (23 mg/mL), respectively; another group was treated with TCF-ether (10 μM) in DMEM which was added with or without BSA (23 mg/mL), respectively. The cells were incubated for 2 h at 37 °C and then washed three times with a PBS buffer before fluorescence imaging.

For the dose-dependent experiment, the cells were first incubated with a probe system (TCF-ether + PdCl_2_) for 30 min at 37°C. They were then treated with CORM-2 at a final concentration of 0 μM, 20 μM, and 40 μM respectively, for another 30 min at 37 °C. After washing three times with PBS, fluorescence images of the cells were captured.

### 3.5. Molecular Docking

The crystal structure of HSA (PDB id: 2BXG) and of BSA (PDB id: 4F5S) were taken from Brookhaven Protein Data Bank and the 3D structure of DCI-OH and TCF-OH was obtained from OpenBabel. The docking studies were performed by auto dock 4.2.0 software [66]. Lamarckian genetic algorithm (LGA) implemented with an adaptive local method search was applied to rule out the possible conformation of DCI-OH and TCF-OH that binds to the protein. Hydrogen atoms and water molecules were eliminated, and then partial Kollman charges were designated to the proteins (HSA and BSA). The proteins were set to be rigid and all the torsional bonds were taken as being free during the docking process. The solvent molecules were not considered during docking. To reveal the binding site of DCI-OH and TCF-OH on HSA and BSA, docking was performed and the grid size was set to be two times the size of ibuprofen for 2BXG. Auto dock parameters were used with 150 as GA population size and 2,500,000 as a maximum number of energy evolutions. The ten best solutions based on docking score were retained for further investigations. PyMOL 2.4 was used to visualize and recognize the residues involved in the binding process.

## 4. Conclusions

In summary, we constructed a novel fluorescent probe system applying allyl ether for CO detection, which can avoid the effect of BSA. The probe system is readily available and able to detect CO under physiological conditions. TCF-ether can be used to detect CO with excellent sensing properties including high selectivity and sensitivity. It should be noted that TCF-ether has more glorious stability in BSA than TCF-ester and a rapid response to CO within 15 min. In addition, fluorescence probe TCF-ether with a long wavelength (618 nm) is particularly useful in practical applications. The probe system has been successfully applied to detect CO in living cells. More importantly, utilizing an allyl ether to avoid problems caused by hydrolysis could also inspire other areas such as pharmacy and medical imaging.

## Figures and Tables

**Table 1:** Molecular docking parameters were obtained from Albumin-DCI-OH or TCF-OH interaction.

Host	Guest	Amino Acid Residues	Forces Involved	ΔG (kcal·mol^−1^)
BSA	DCI-OH	SER201, PHE205, ARG208, ALA209, ALA212, TRP213, ASP323, LEU326, PHE329, LEU330, SER343, LEU346, GLU353, SER453,	Hydrophobic interaction	−8.3
		GLY327, LYS350, LEU480	Hydrogen bond	
	TCF-OH	L197, F205, R208, A209, A212, W213, D323, L326, G327, L330, L346, A349, K350, E353, S453, T477, E478, S479, L480, V481	Hydrophobic interaction	−6.2
			Hydrogen bond	
HSA	DCI-OH	L198, S202, F206, A210, A213, W214, V216, D324, L327, G328, L331, V343, L347, A350, K351, E354, P384, S454, S480, L481, V482, N483	Hydrophobic interaction	−9.3
		R209	Hydrogen bond	
	TCF-OH	L198, S202, F206, R209, A210, A213, W214, L327, G328, L331, L347, A350, K351, E354, S454, T478, E479, S480, N483	Hydrophobic interaction	−8.7
		D324, L481, V482	Hydrogen bond	

## Data Availability

Data supporting reported results are available online.

## Supplementary Materials

The following supporting information is available online at: https://www.mdpi.com/article/10.3390/molecules27134155/s1, Figure S1: ^1^H-NMR and ^13^C-NMR of DCI-ether; Figure S2: ^1^H-NMR and ^13^C-NMR of TCF-ether; Figure S3. ^1^H-NMR and ^13^C-NMR of TCF-OH; Figure S4. ^1^H-NMR and ^13^C-NMR of TCF-ester; Figure S5. ^1^H-NMR and ^13^C-NMR of TCF; Figure S6. ^1^H-NMR of DCI; Figure S7. ^1^H-NMR and ^13^C-NMR of DCI-OH; Figure S8. ^1^H-NMR and ^13^C-NMR of DCI-ester.

## Sample Availability

Samples of the compounds TCF-ether are available from the authors.

## References

[1] Omaye S.T. (2002). Metabolic Modulation of Carbon Monoxide Toxicity. Toxicology.

[2] Schatzschneider U. (2015). Novel Lead Structures and Activation Mechanisms for CO-Releasing Molecules (CORMs): CO-Releasing Molecules (CORMs). Br. J. Pharmacol..

[3] Scragg J.L., Dallas M.L., Wilkinson J.A., Varadi G., Peers C. (2008). Carbon Monoxide Inhibits L-Type Ca^2+^ Channels via Redox Modulation of Key Cysteine Residues by Mitochondrial Reactive Oxygen Species. J. Biol. Chem..

[4] Tenhunen R., Marver H.S., Schmid R. (1968). The Enzymatic Conversion of Heme to Bilirubin by Microsomal Heme Oxygenase. Proc. Natl. Acad. Sci. USA.

[5] Ryter S.W., Alam J., Choi A.M.K. (2006). Heme Oxygenase-1/Carbon Monoxide: From Basic Science to Therapeutic Applications. Physiol. Rev..

[6] Li Y., Wang X., Yang J., Xie X., Li M., Niu J., Tong L., Tang B. (2016). Fluorescent Probe Based on Azobenzene-Cyclopalladium for the Selective Imaging of Endogenous Carbon Monoxide under Hypoxia Conditions. Anal. Chem..

[7] Heme Oxygenase-1/Carbon Monoxide: From Basic Science to Therapeutic Applications|Physiological Reviews. https://journals.physiology.org/doi/full/10.1152/physrev.00011.2005.

[8] Gu Y.-J., Cheng J., Man C.W.-Y., Wong W.-T., Cheng S.H. (2012). Gold-Doxorubicin Nanoconjugates for Overcoming Multidrug Resistance. Nanomed. Nanotechnol. Biol. Med..

[9] Overexpression of HO-1 Protects against TNF-α-Mediated Airway Inflammation by Down-Regulation of TNFR1-Dependent Oxidative Stress-ScienceDirect. https://www.sciencedirect.com/science/article/pii/S0002944010605676.

[10] Clark J.E., Naughton P., Shurey S., Green C.J., Johnson T.R., Mann B.E., Foresti R., Motterlini R. (2003). Cardioprotective Actions by a Water-Soluble Carbon Monoxide-Releasing Molecule. Circ. Res..

[11] Insertable Fast-Response Amperometric NO/CO Dual Microsensor: Study of Neurovascular Coupling during Acutely Induced Seizures of Rat Brain Cortex|Analytical Chemistry. https://pubs.acs.org/doi/10.1021/acs.analchem.5b04288.

[12] Paredi P., Biernacki W., Invernizzi G., Kharitonov S.A., Barnes P.J. (1999). Exhaled Carbon Monoxide Levels Elevated in Diabetes and Correlated with Glucose Concentration in Blood: A New Test for Monitoring the Disease?. Chest.

[13] Curcumin Activates the Haem Oxygenase-1 Gene via Regulation of Nrf2 and the Antioxidant-Responsive Element|Biochemical Journal|Portland Press. https://portlandpress.com/biochemj/article-abstract/371/3/887/40597/Curcumin-activates-the-haem-oxygenase-1-gene-via?redirectedFrom=fulltext.

[14] Zhou X., Lee S., Xu Z., Yoon J. (2015). Recent Progress on the Development of Chemosensors for Gases. Chem. Rev..

[15] Yamashita K., McDaid J., Ollinger R., Tsui T.-Y., Berberat P.O., Usheva A., Csizmadia E., Smith R.N., Soares M.P., Bach F.H. (2004). Biliverdin, a Natural Product of Heme Catabolism, Induces Tolerance to Cardiac Allografts. FASEB J. Off. Publ. Fed. Am. Soc. Exp. Biol..

[16] Morita T., Mitsialis S.A., Koike H., Liu Y., Kourembanas S. (1997). Carbon Monoxide Controls the Proliferation of Hypoxic Vascular Smooth Muscle Cells. J. Biol. Chem..

[17] Neuroprotective, Neurotherapeutic, and Neurometabolic Effects of Carbon Monoxide-PubMed. https://pubmed.ncbi.nlm.nih.gov/23270619/.

[18] Benito-Garagorri D., Puchberger M., Mereiter K., Kirchner K. (2008). Stereospecific and Reversible CO Binding at Iron Pincer Complexes. Angew. Chem. Int. Ed..

[19] Heylen S., Martens J.A. (2010). Progress in the Chromogenic Detection of Carbon Monoxide. Angew. Chem. Int. Ed..

[20] Sensitive and Selective Chromogenic Sensing of Carbon Monoxide via Reversible Axial CO Coordination in Binuclear Rhodium Complexes|Journal of the American Chemical Society. https://pubs.acs.org/doi/10.1021/ja206251r.

[21] Park S.S., Kim J., Lee Y. (2012). Improved Electrochemical Microsensor for the Real-Time Simultaneous Analysis of Endogenous Nitric Oxide and Carbon Monoxide Generation. Anal. Chem..

[22] Lee Y., Kim J. (2007). Simultaneous Electrochemical Detection of Nitric Oxide and Carbon Monoxide Generated from Mouse Kidney Organ Tissues. Anal. Chem..

[23] Chen X., Wang F., Hyun J.Y., Wei T., Qiang J., Ren X., Shin I., Yoon J. (2016). Recent Progress in the Development of Fluorescent, Luminescent and Colorimetric Probes for Detection of Reactive Oxygen and Nitrogen Species. Chem. Soc. Rev..

[24] Wu D., Sedgwick A.C., Gunnlaugsson T., Akkaya E.U., Yoon J., James T.D. (2017). Fluorescent Chemosensors: The Past, Present and Future. Chem. Soc. Rev..

[25] Du J., Hu M., Fan J., Peng X. (2012). Fluorescent Chemodosimeters Using “Mild” Chemical Events for the Detection of Small Anions and Cations in Biological and Environmental Media. Chem. Soc. Rev..

[26] Mukhopadhyay S., Sarkar A., Chattopadhyay P., Dhara K. (2020). Recent Advances in Fluorescence Light-Up Endogenous and Exogenous Carbon Monoxide Detection in Biology. Chem.-Asian J..

[27] Tikum A.F., Lim W., Fortibui M.M., Lee S., Park S., Kim J. (2021). Palladium Probe Consisting of Naphthalimide and Ethylenediamine for Selective Turn-On Sensing of CO and Cell Imaging. Inorg. Chem..

[28] Shi G., Yoon T., Cha S., Kim S., Yousuf M., Ahmed N., Kim D., Kang H.-W., Kim K.S. (2018). Turn-on and Turn-off Fluorescent Probes for Carbon Monoxide Detection and Blood Carboxyhemoglobin Determination. ACS Sens..

[29] Wang J., Karpus J., Zhao B.S., Luo Z., Chen P.R., He C. (2012). A Selective Fluorescent Probe for Carbon Monoxide Imaging in Living Cells. Angew. Chem. Int. Ed..

[30] Gong S., Hong J., Zhou E., Feng G. (2019). A Near-Infrared Fluorescent Probe for Imaging Endogenous Carbon Monoxide in Living Systems with a Large Stokes Shift. Talanta.

[31] Wang J., Li C., Chen Q., Li H., Zhou L., Jiang X., Shi M., Zhang P., Jiang G., Tang B.Z. (2019). An Easily Available Ratiometric Reaction-Based AIE Probe for Carbon Monoxide Light-up Imaging. Anal. Chem..

[32] Michel B.W., Lippert A.R., Chang C.J. (2012). A Reaction-Based Fluorescent Probe for Selective Imaging of Carbon Monoxide in Living Cells Using a Palladium-Mediated Carbonylation. J. Am. Chem. Soc..

[33] Robson J.A., Kubánková M., Bond T., Hendley R.A., White A.J.P., Kuimova M.K., Wilton-Ely J.D.E.T. (2020). Simultaneous Detection of Carbon Monoxide and Viscosity Changes in Cells. Angew. Chem..

[34] Toscani A., Marín-Hernández C., Robson J.A., Chua E., Dingwall P., White A.J.P., Sancenón F., de la Torre C., Martínez-Máñez R., Wilton-Ely J.D.E.T. (2019). Highly Sensitive and Selective Molecular Probes for Chromo-Fluorogenic Sensing of Carbon Monoxide in Air, Aqueous Solution and Cells. Chem.-Eur. J..

[35] Marín-Hernández C., Toscani A., Sancenón F., Wilton-Ely J.D.E.T., Martínez-Máñez R. (2016). Chromo-Fluorogenic Probes for Carbon Monoxide Detection. Chem. Commun..

[36] Wang H. (2010). Tsuji-Trost Reaction: (Tsuji-Trost Allylation, Tsuji-Trost Allylic Substitution, Tsuji Allylation). Comprehensive Organic Name Reactions and Reagents.

[37] Li J.J., Li J.J. (2014). Tsuji–Trost Reaction. Name Reactions: A Collection of Detailed Mechanisms and Synthetic Applications Fifth Edition.

[38] Li J.J., Pelletier S.W. (1999). Chapter Three—Applications of Palladium Chemistry to the Total Syntheses of Naturally Occurring Indole Alkaloids. Alkaloids: Chemical and Biological Perspectives.

[39] Feng W., Feng G. (2018). A Readily Available Colorimetric and Near-Infrared Fluorescent Turn-on Probe for Detection of Carbon Monoxide in Living Cells and Animals. Sens. Actuators B Chem..

[40] Feng S., Liu D., Feng W., Feng G. (2017). Allyl Fluorescein Ethers as Promising Fluorescent Probes for Carbon Monoxide Imaging in Living Cells. Anal. Chem..

[41] Feng W., Liu D., Feng S., Feng G. (2016). Readily Available Fluorescent Probe for Carbon Monoxide Imaging in Living Cells. Anal. Chem..

[42] Feng W., Bai L., Jia S., Feng G. (2018). A Novel Phthalimide-Rhodol-Based ESIPT-FRET System for Rapid Colorimetric and Ratiometric Fluorescent Detection of Palladium. Sens. Actuators B Chem..

[43] Xia Q., Feng S., Liu D., Feng G. (2018). A Highly Selective and Sensitive Colorimetric and Near-Infrared Fluorescent Turn-on Probe for Rapid Detection of Palladium in Drugs and Living Cells. Sens. Actuators B Chem..

[44] Yan J., Zhu J., Tan Q., Zhou L., Yao P., Lu Y., Tan J., Zhang L. (2016). Development of a Colorimetric and NIR Fluorescent Dual Probe for Carbon Monoxide. RSC Adv..

[45] Xu Z., Yan J., Li J., Yao P., Tan J., Zhang L. (2016). A Colorimetric and Fluorescent Turn-on Probe for Carbon Monoxide and Imaging in Living Cells. Tetrahedron Lett..

[46] Rabbani G., Ahn S.N. (2019). Structure, Enzymatic Activities, Glycation and Therapeutic Potential of Human Serum Albumin: A Natural Cargo. Int. J. Biol. Macromol..

[47] Salvi A., Carrupt P.-A., Mayer J.M., Testa B. (1997). Esterase-like Activity of Human Serum Albumin Toward Prodrug Esters of Nicotinic Acid. Drug Metab. Dispos..

[48] Yang F., Bian C., Zhu L., Zhao G., Huang Z., Huang M. (2007). Effect of Human Serum Albumin on Drug Metabolism: Structural Evidence of Esterase Activity of Human Serum Albumin. J. Struct. Biol..

[49] Sun Q., Wang W., Chen Z., Yao Y., Zhang W., Duan L., Qian J. (2017). A Fluorescence Turn-on Probe for Human (Bovine) Serum Albumin Based on the Hydrolysis of a Dioxaborine Group Promoted by Proteins. Chem. Commun..

[50] Jin Q., Feng L., Zhang S.-J., Wang D.-D., Wang F.-J., Zhang Y., Cui J.-N., Guo W.-Z., Ge G.-B., Yang L. (2017). Real-Time Tracking the Synthesis and Degradation of Albumin in Complex Biological Systems with a near-Infrared Fluorescent Probe. Anal. Chem..

[51] Ge G.-B., Feng L., Jin Q., Wang Y.-R., Liu Z.-M., Zhu X.-Y., Wang P., Hou J., Cui J.-N., Yang L. (2017). A Novel Substrate-Inspired Fluorescent Probe to Monitor Native Albumin in Human Plasma and Living Cells. Anal. Chim. Acta.

[52] Kim S.-J., Rhee H.-W., Park H.-J., Kim H.-Y., Kim H.-S., Hong J.-I. (2013). Fluorescent Probes Designed for Detecting Human Serum Albumin on the Basis of Its Pseudo-Esterase Activity. Bioorg. Med. Chem. Lett..

[53] Ma D.H., Kim D., Akisawa T., Lee K.-H., Kim K.-T., Ahn K.H. (2015). An FITC-BODIPY FRET Couple: Application to Selective, Ratiometric Detection and Bioimaging of Cysteine. Chem.-Asian J..

[54] Yang Y.J., Dai M., Reo Y.J., Song C.W., Sarkar S., Ahn K.H. (2021). NAD(P)H Quinone Oxidoreductase-1 in Organ and Tumor Tissues: Distinct Activity Levels Observed with a Benzo-Rosol-Based Dual-Excitation and Dual-Emission Probe. Anal. Chem..

[55] Yoon J.W., Kim S., Yoon Y., Lee M.H. (2019). A Resorufin-Based Fluorescent Turn-on Probe Responsive to Nitroreductase Activity and Its Application to Bacterial Detection. Dye. Pigment..

[56] Xu J., Wang Z., Liu C., Xu Z., Wang N., Cong X., Zhu B. (2018). A Highly Selective Colorimetric and Long-Wavelength Fluorescent Probe for the Detection of Hg^2+^. Luminescence.

[57] Gopalan P., Katz H.E., McGee D.J., Erben C., Zielinski T., Bousquet D., Muller D., Grazul J., Olsson Y. (2004). Star-Shaped Azo-Based Dipolar Chromophores: Design, Synthesis, Matrix Compatibility, and Electro-Optic Activity. J. Am. Chem. Soc..

[58] Lord S.J., Conley N.R., Lee H.D., Samuel R., Liu N., Twieg R.J., Moerner W.E. (2008). A Photoactivatable Push−Pull Fluorophore for Single-Molecule Imaging in Live Cells. J. Am. Chem. Soc..

[59] Lee M.K., Williams J., Twieg R.J., Rao J., Moerner W.E. (2013). Enzymatic Activation of Nitro-Aryl Fluorogens in Live Bacterial Cells for Enzymatic Turnover-Activated Localization Microscopy. Chem. Sci..

[60] Zhang W., Huo F., Yin C. (2018). Recent Advances of Dicyano-Based Materials in Biology and Medicine. J. Mater. Chem. B.

[61] Teng X., Tian M., Zhang J., Tang L., Xin J. (2018). A TCF-Based Colorimetric and Fluorescent Probe for Palladium Detection in an Aqueous Solution. Tetrahedron Lett..

[62] Wang Z., Zhao Z., Liu C., Geng Z., Duan Q., Jia P., Li Z., Zhu H., Zhu B., Sheng W. (2019). A Long-Wavelength Ultrasensitive Colorimetric Fluorescent Probe for Carbon Monoxide Detection in Living Cells. Photochem. Photobiol. Sci..

[63] Liyasova M.S., Schopfer L.M., Lockridge O. (2010). Reaction of Human Albumin with Aspirin in Vitro: Mass Spectrometric Identification of Acetylated Lysines 199, 402, 519, and 545. Biochem. Pharmacol..

[64] Bak I., Szendrei L., Turoczi T., Papp G., Joo F., Das D.K., de Leiris J., Der P., Juhasz B., Varga E. (2003). Heme Oxygenase-1-Related Carbon Monoxide Production and Ventricular Fibrillation in Isolated Ischemic/Reperfused Mouse Myocardium. FASEB J. Off. Publ. Fed. Am. Soc. Exp. Biol..

[65] Wu H., Yang X., Men J., Zhang H., Zhou J. (2019). A Near-Infrared Fluorescent Probe of Dicyanoisophorone Derivatives for Selective Detection and Fluorescence Cellular Imaging of Palladium. Anal. Sci..

[66] Trott O., Olson A.J. (2009). AutoDock Vina: Improving the Speed and Accuracy of Docking with a New Scoring Function, Efficient Optimization, and Multithreading. J. Comput. Chem..

