# Supplementary material for: A TCF-Based Carbon Monoxide NIR-Probe without the Interference of BSA and Its Application in Living Cells
Source: Molecules. 2022 Jun 28;27(13):4155. doi: 10.3390/molecules27134155 (PMC9268636; doi:10.3390/molecules27134155)
Supplement: Supplementary file 1 [file molecules-27-04155-s001.zip › molecules-1794217-supplementary.pdf]

Supplementary Materials

A TCF-Based Carbon Monoxide NIR-Probe without the Interference of BSA and Its Application in Living Cells

Yingxu Wu [1], Xiaojing Deng [1], Lan Ye [2], Wei Zhang [3], Hu Xu [2,*] and Boyu Zhang [1,*]

1 College of Medical Laboratory, Dalian Medical University, Dalian 116044, China; wuyingux1111@gmail.com (Y.W.); dengxiaojing0728@hotmail.com (X.D.)
2 Advanced Institute for Medical Sciences, Dalian Medical University, Dalian 116044, China; 15606072750@163.com
3 Department of Spine Surgery, The Second Hospital of Dalian Medical University, Dalian 116023, China; zhangwei1983@dmu.edu.cn
* Correspondence: xuhu@dmu.edu.cn (H.X.); byzhang@dmu.edu.cn (B.Z.)

^{1}H-NMR and ^{13}C-NMR analysis reports of TCF, DCI，DCI-OH, TCF-OH, DCI-ester, TCF-ester, DCI-ether and TCF-ether.

TCF: ^{1}H-NMR (400 MHz, DMSO-d6) δ 2.37 (s, 3H), 1.60 (s, 6H). ^{13}C-NMR (101 MHz, DMSO) δ 186.22, 177.75, 112.69, 111.97, 110.44, 104.10, 101.81, 55.24, 39.66, 23.69, 14.68.

TCF-OH: ^{1}H-NMR (400 MHz, DMSO-d6) δ 10.59 (s, 1H), 7.90 (d, *J* = 16.2 Hz, 1H), 7.81 (d, *J* = 8.2 Hz, 2H), 7.02 (d, *J* = 16.1 Hz, 1H), 6.90 (d, *J* = 8.2 Hz, 2H), 1.78 (s, 5H). ^{13}C-NMR (101 MHz, DMSO) δ 177.70, 176.32, 162.77, 148.81, 132.79, 126.17, 116.93, 113.39, 112.56, 112.19, 111.70, 99.48, 97.03, 53.71, 39.87, 39.67, 39.47, 25.78.

DCI-OH: ^{1}H-NMR (400 MHz, DMSO-d6) δ 10.06 (s, 1H), 7.67-7.59 (m, 2H), 7.36-7.21 (m, 2H), 6.91-6.83 (m, 3H), 2.67 (s, 2H), 2.60 (s, 2H), 1.09 (s, 6H). ^{13}C-NMR (101 MHz, DMSO) δ 165.67, 155.12, 115.80, 108.44, 107.66, 73.42, 72.64, 72.53, 72.32, 72.01, 40.91, 37.88, 27.62, 23.07, 23.05, 20.57.

DCI: ^{1}H-NMR (400 MHz, Chloroform-d) δ 6.62 (t, *J* = 1.7 Hz, 1H), 2.51 (d, *J* = 1.4 Hz, 2H), 2.17 (s, 2H), 2.03 (s, 3H), 1.01 (d, *J* = 1.4 Hz, 6H).

TCF-ester: ^{1}H-NMR (400 MHz, DMSO-d6) δ 8.10-8.03 (m, 2H), 7.99 (d, *J* = 16.5 Hz, 1H), 7.56-7.37 (m, 2H), 7.34-7.16 (m, 1H), 6.07 (ddt, *J* = 16.4, 10.9, 5.6 Hz, 1H), 5.53-5.33 (m, 2H), 4.85-4.74 (m, 2H), 1.86 (s, 5H). ^{13}C-NMR (101 MHz, DMSO) δ 177.53, 175.47, 153.69, 152.74, 146.42, 134.20, 132.84, 132.10, 131.37, 130.07, 122.62, 119.52, 116.18, 113.09, 112.26, 111.23, 100.29, 99.95, 69.47, 65.21, 55.11, 25.54, 23.69.

DCI-ester: ^{1}H-NMR (400 MHz, DMSO-d6) δ 7.83 (dd, *J* = 8.6, 1.7 Hz, 2H), 7.49 (d, *J* = 16.3 Hz, 1H), 7.41-7.32 (m, 3H), 6.97 (s, 1H), 6.13-6.00 (m, 1H), 5.52-5.43 (m, 1H), 5.38 (d, *J* = 10.5 Hz, 1H), 4.80 (d, *J* = 5.3 Hz, 2H), 2.69 (s, 2H), 2.62 (s, 2H), 1.09 (d, *J* = 1.6 Hz, 6H). ^{13}C-NMR (101 MHz, DMSO) δ 170.72, 159.81, 157.16, 138.75, 130.33, 127.57, 126.70, 121.83, 116.35, 114.59, 113.78, 75.27, 42.79, 38.66, 32.13, 27.91.

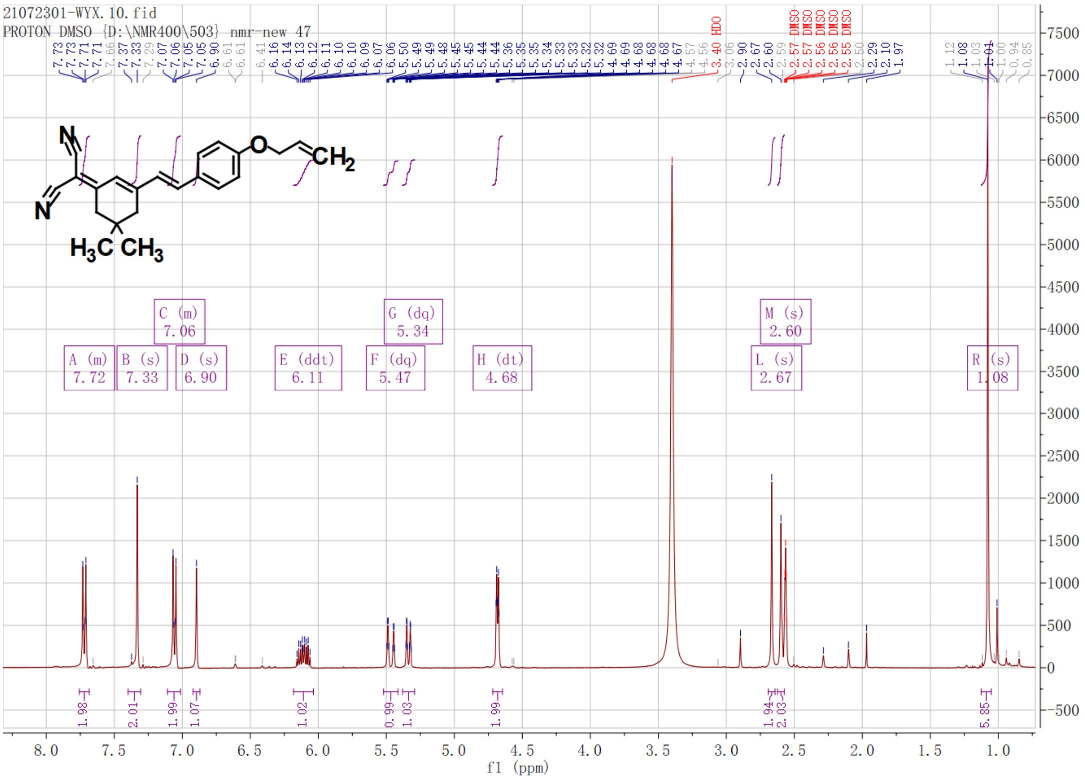

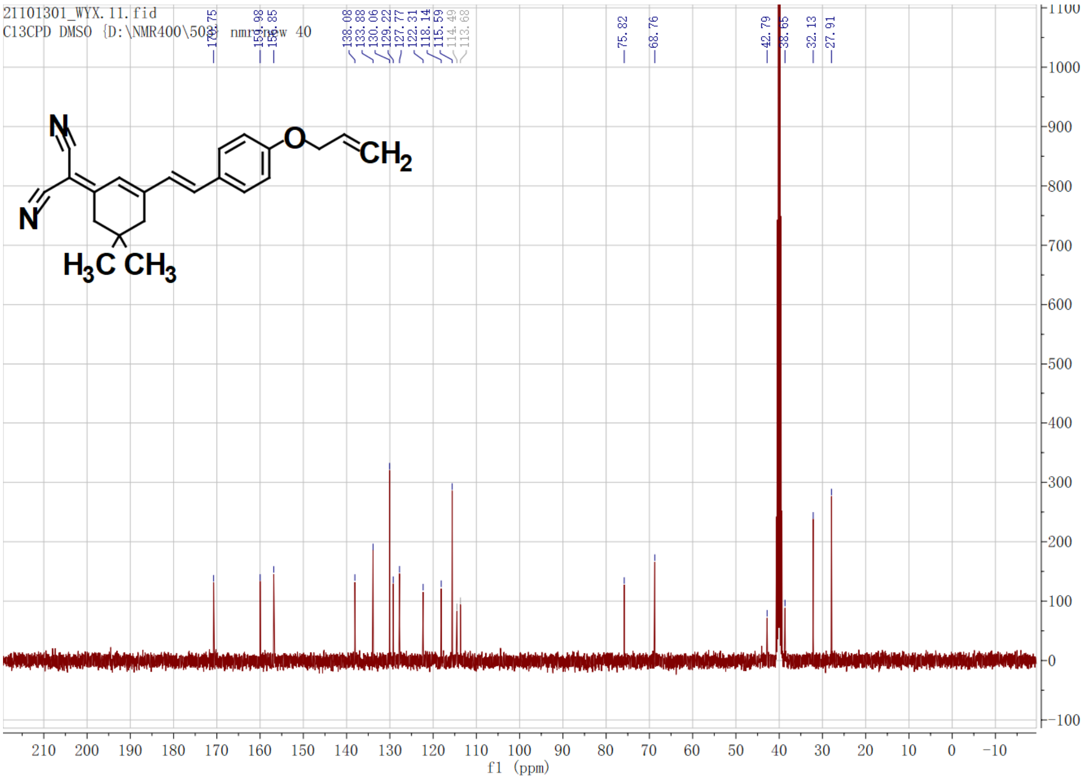

Figure S1. ^1H-NMR and ^{13}C-NMR of DCI-ether.

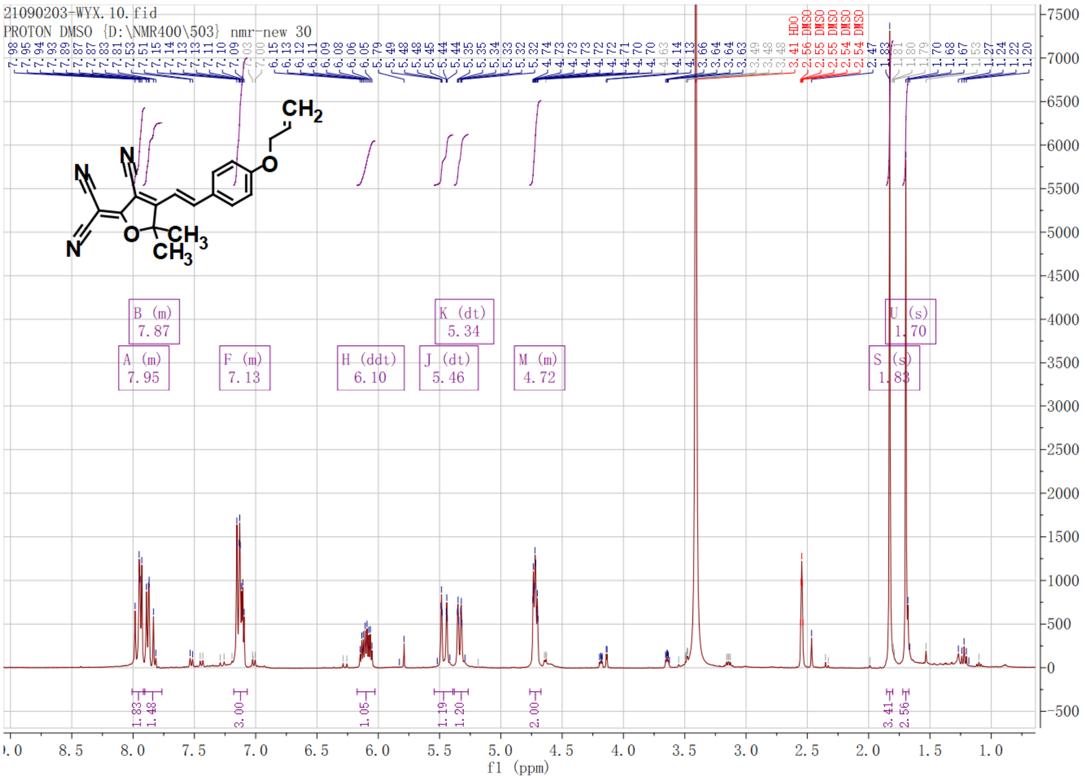

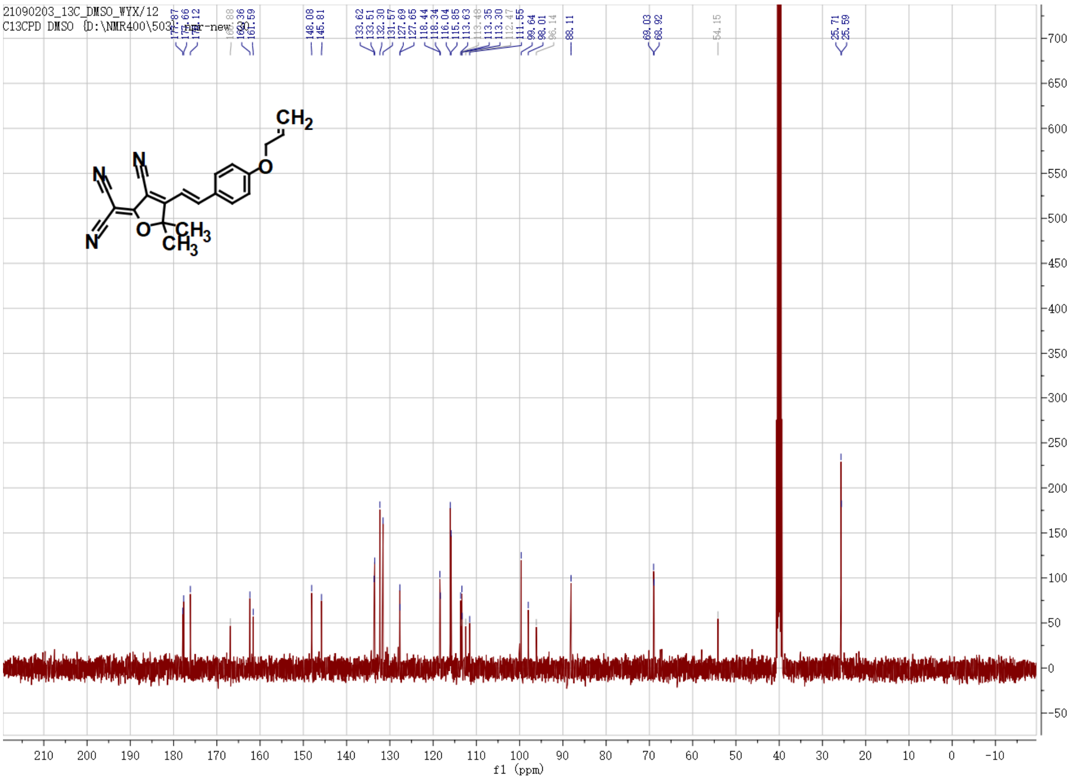

Figure S2. ^{1}H-NMR and ^{13}C-NMR of TCF-ether.

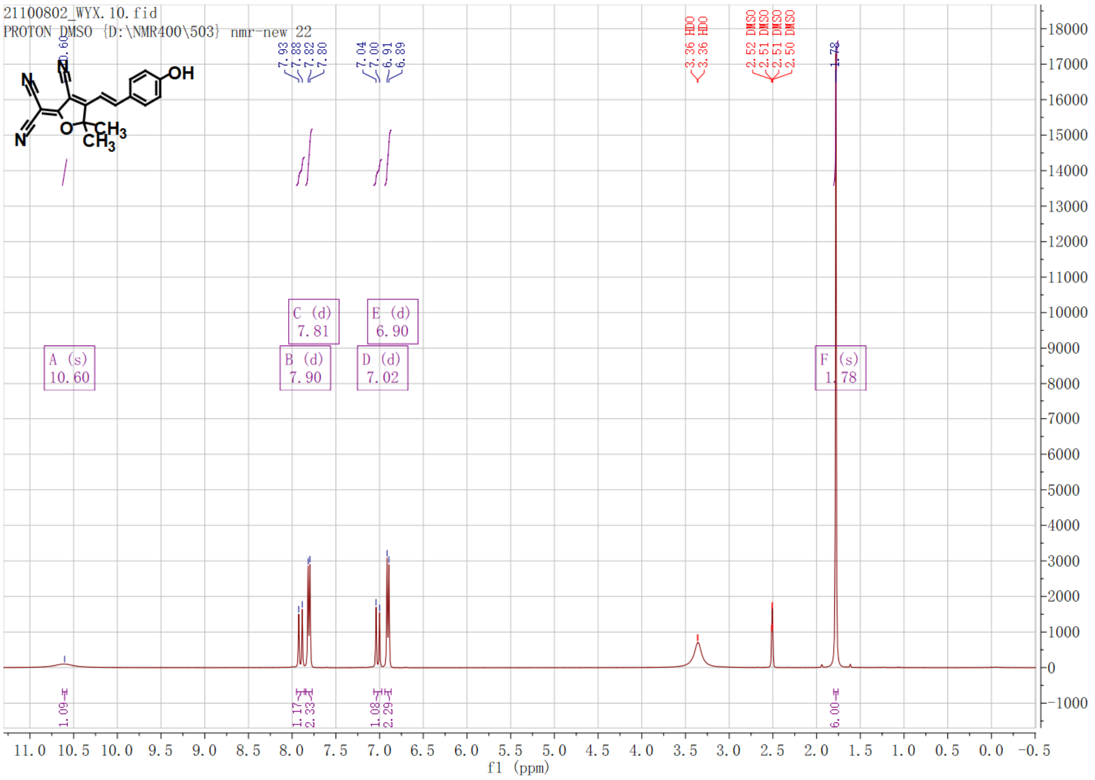

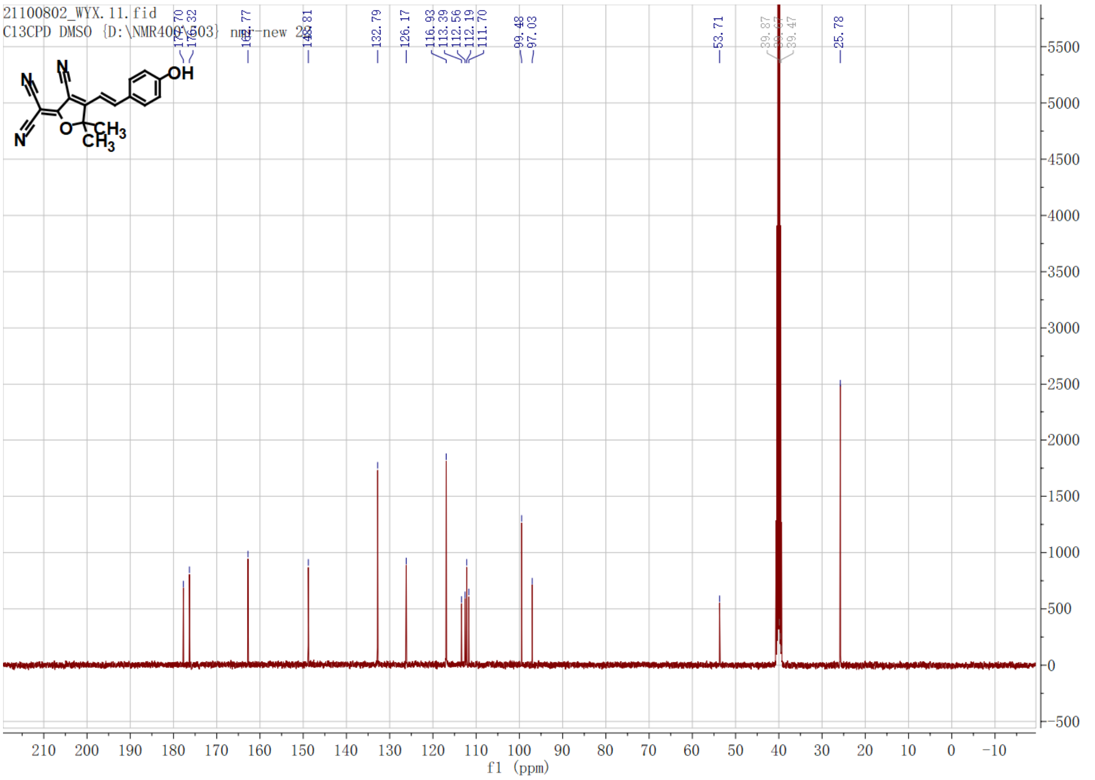

Figure S3. ^{1}H-NMR and ^{13}C-NMR of TCF-OH.

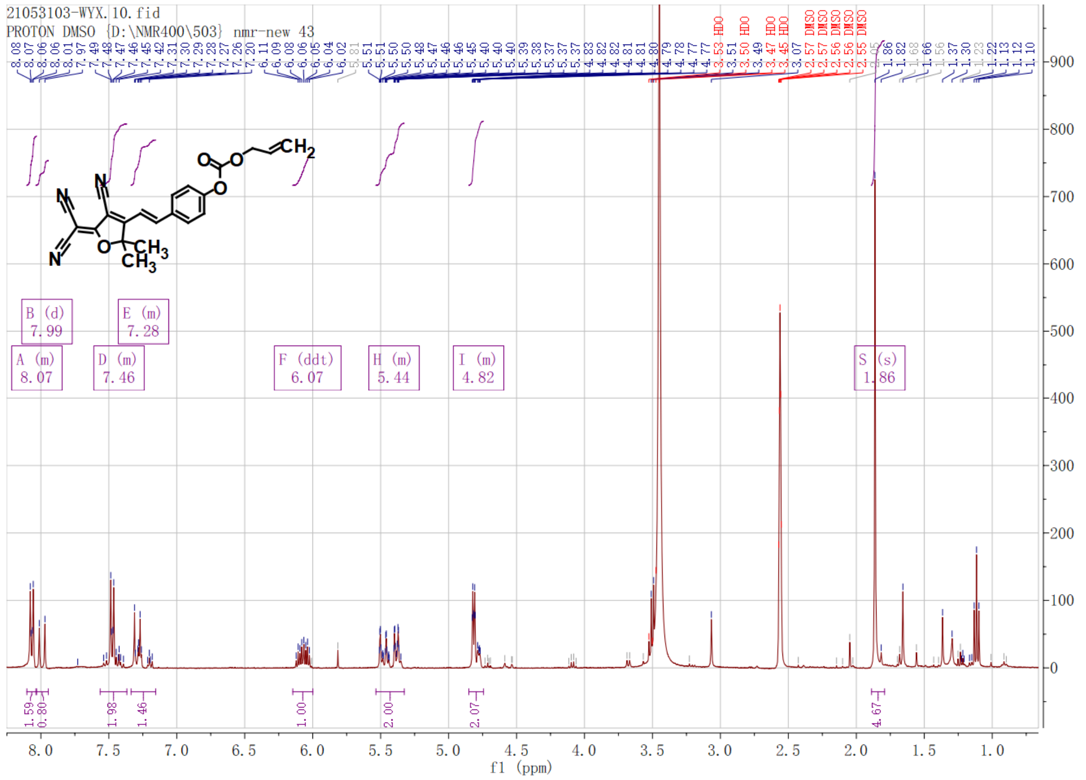

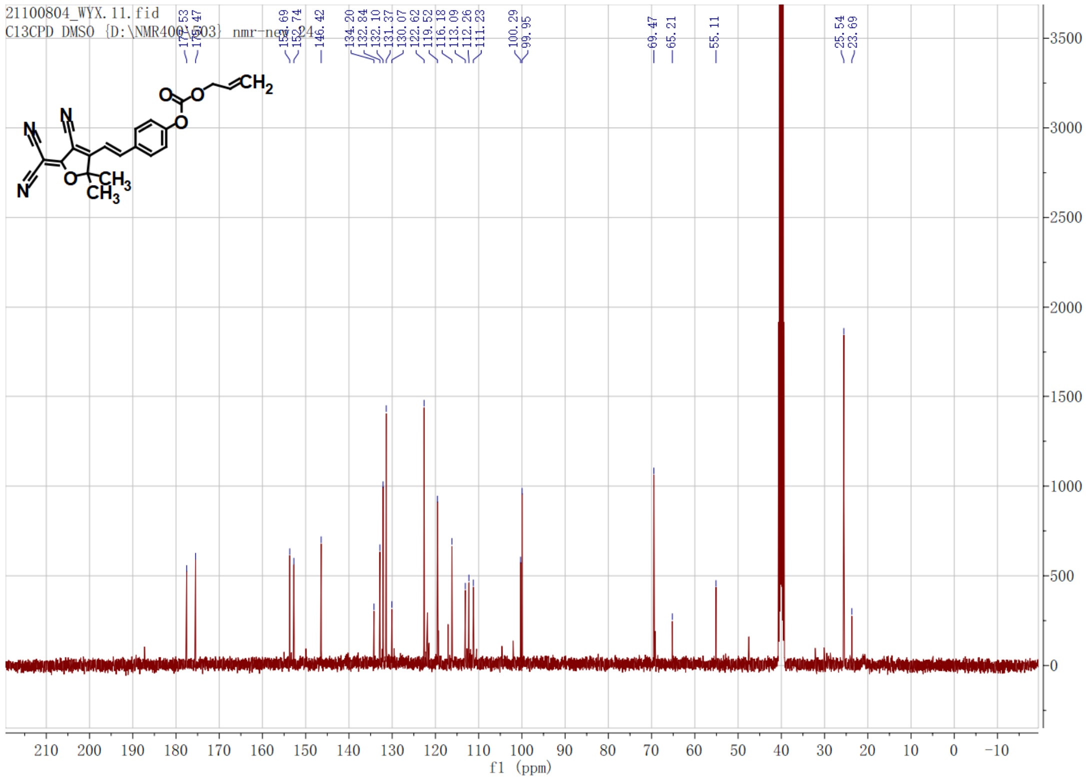

Figure S4. ^{1}H-NMR and ^{13}C-NMR of TCF-ester.

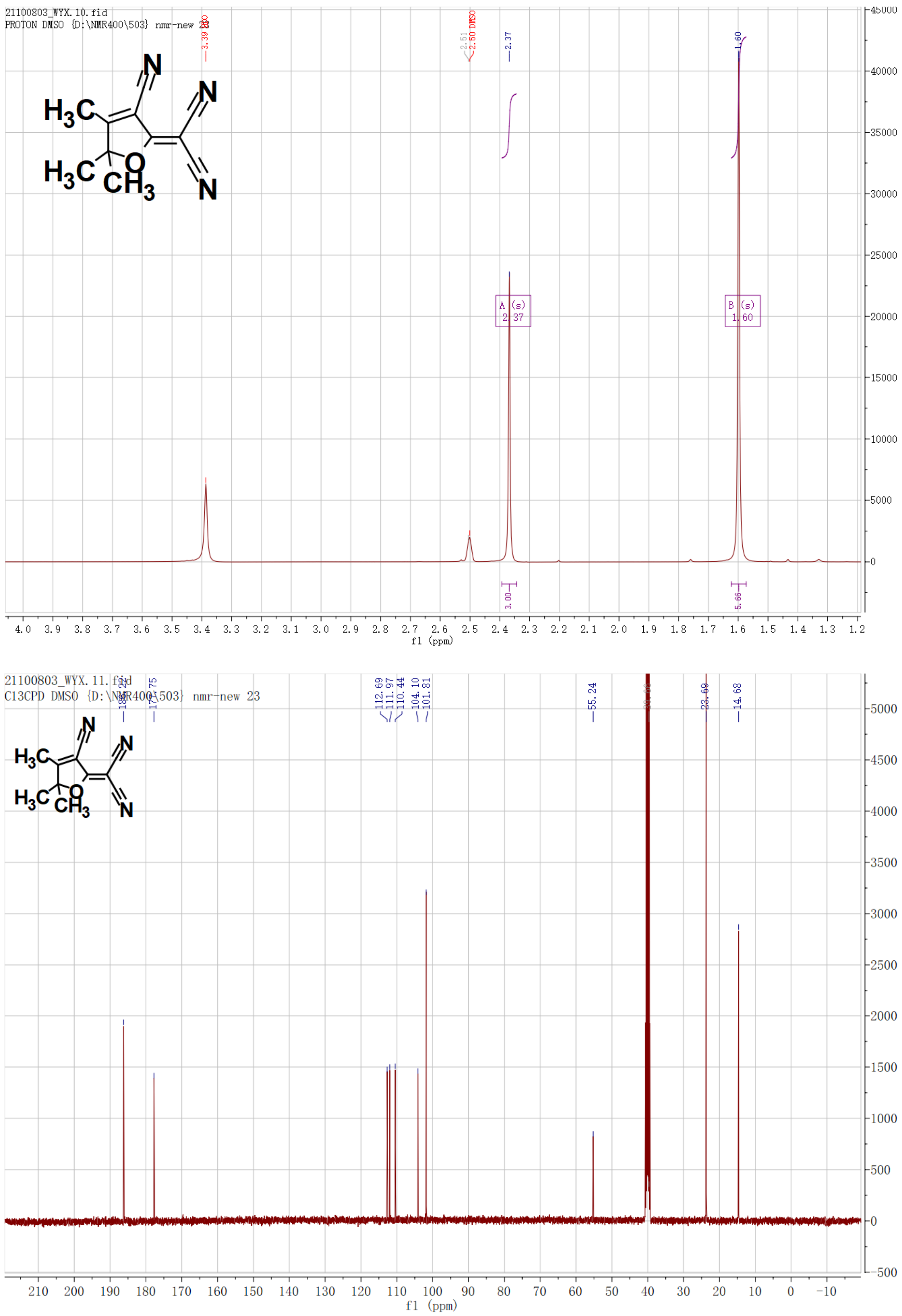

Figure S5. ^{1}H-NMR and ^{13}C-NMR of TCF.

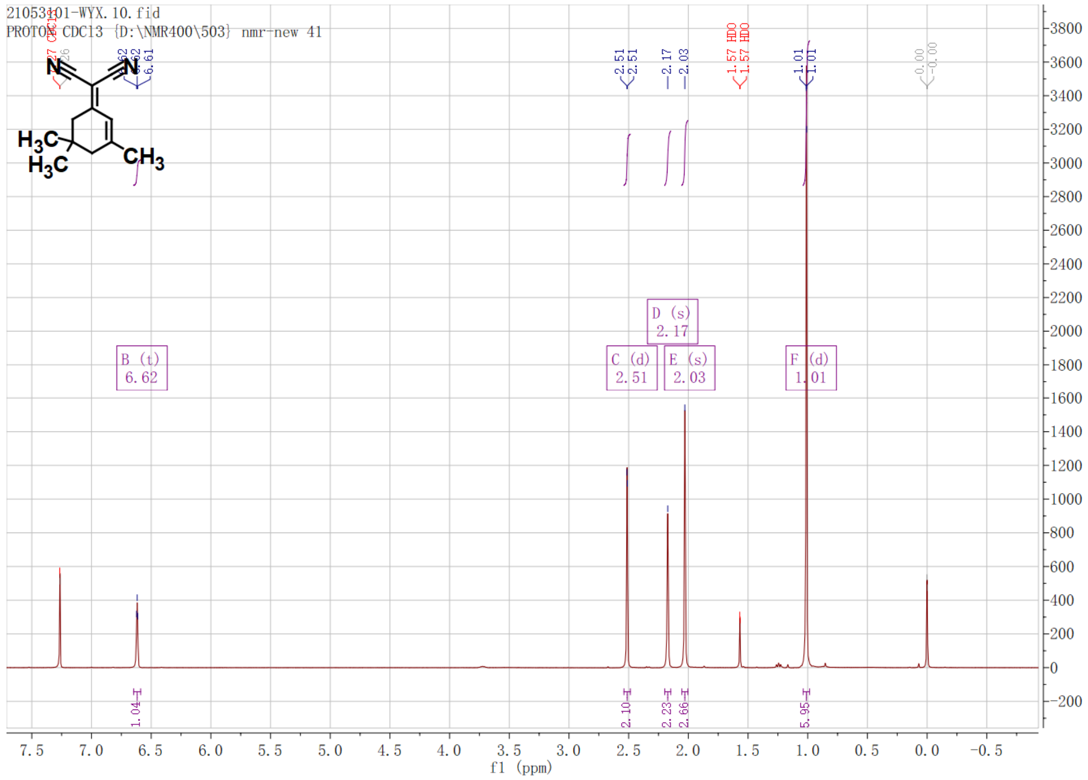

Figure S6. ^{1}H-NMR of DCI.

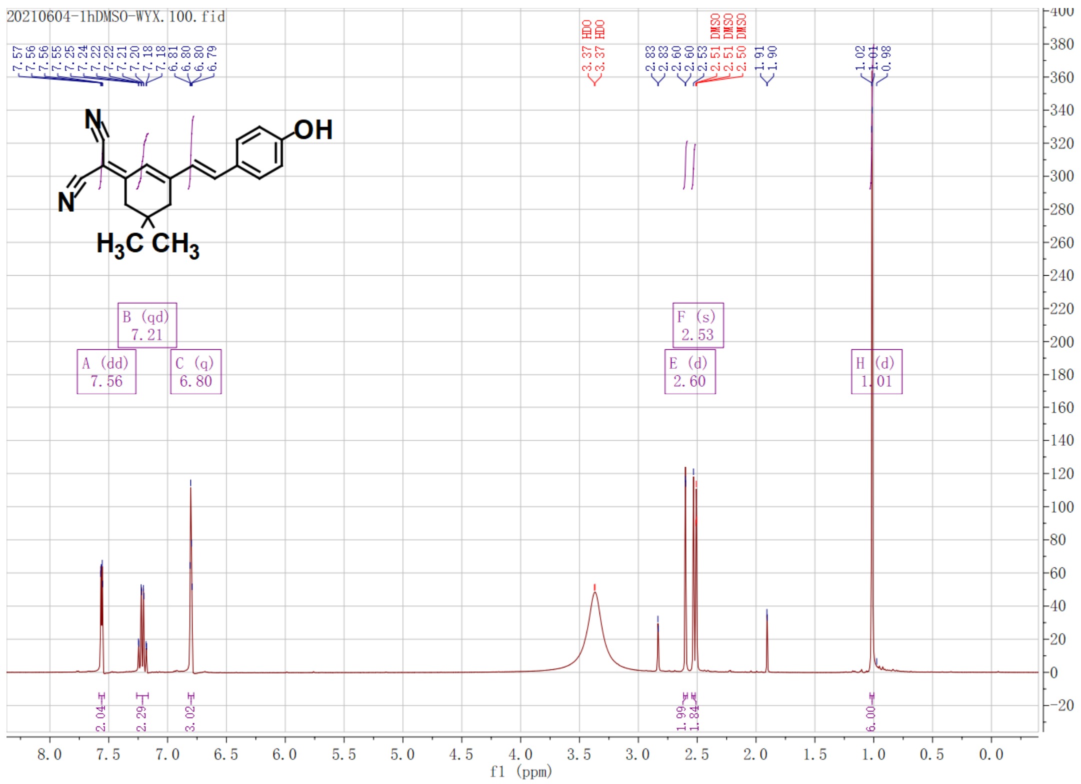

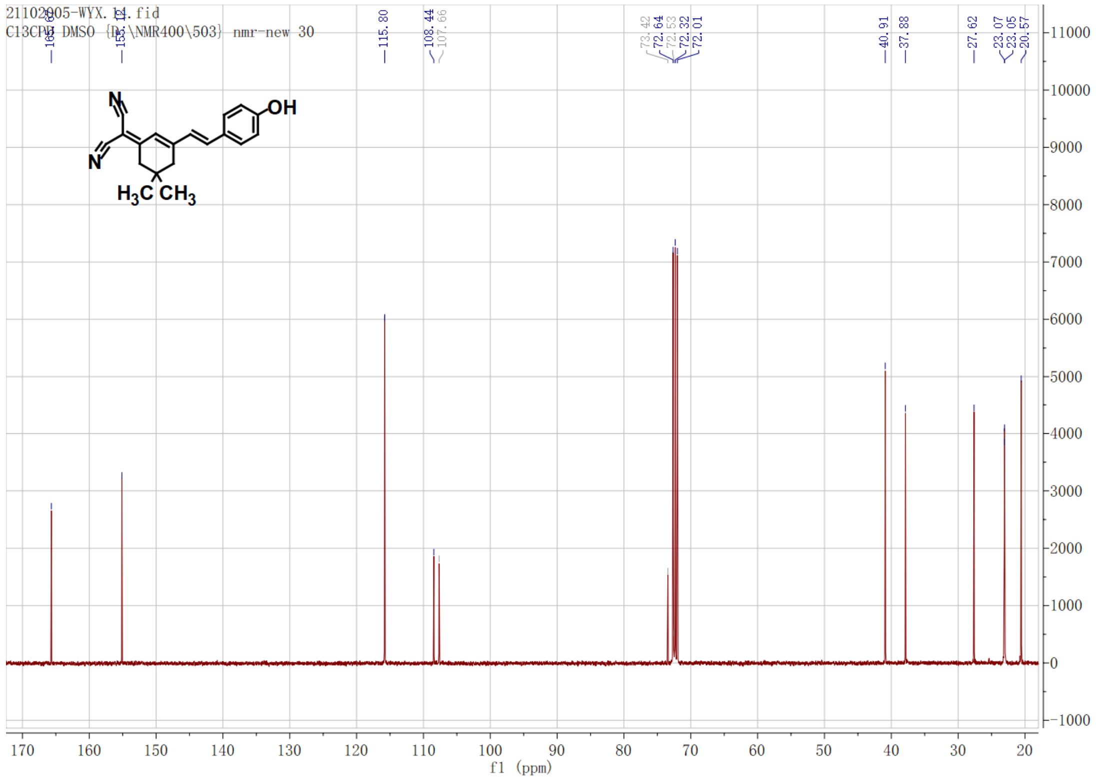

Figure S7. ^{1}H-NMR and ^{13}C-NMR of DCI-OH.

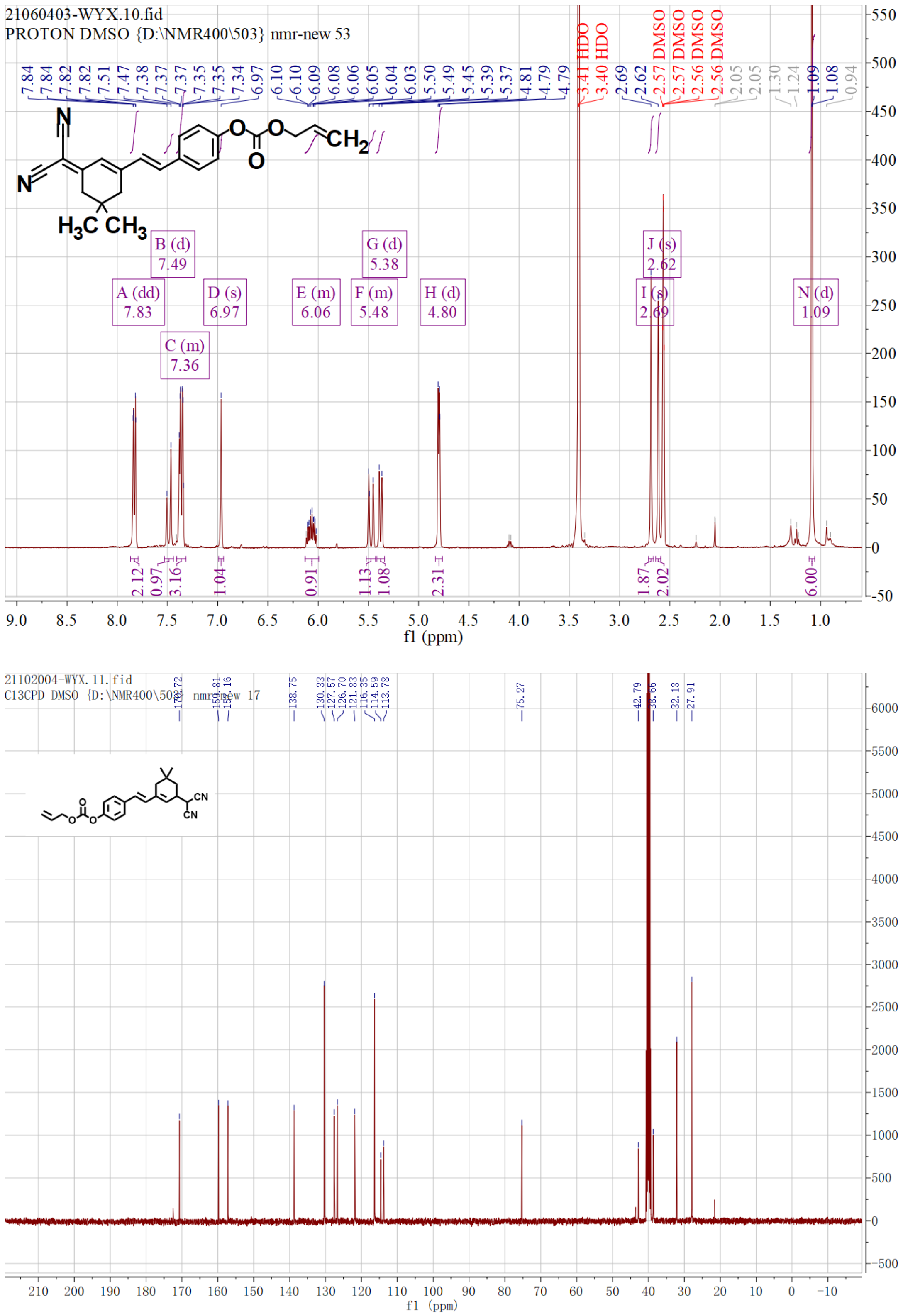

Figure S8. ^{1}H-NMR and ^{13}C-NMR of DCI-ester.
